# ADAM8-Dependent Extracellular Signaling in the Tumor Microenvironment Involves Regulated Release of Lipocalin 2 and MMP-9

**DOI:** 10.3390/ijms23041976

**Published:** 2022-02-10

**Authors:** Lena Cook, Marie Sengelmann, Birte Winkler, Constanze Nagl, Sarah Koch, Uwe Schlomann, Emily P. Slater, Miles A. Miller, Elke Pogge von Strandmann, Bastian Dörsam, Christian Preußer, Jörg W. Bartsch

**Affiliations:** 1 Department of Neurosurgery, Philipps University Marburg, Baldingerstr, 35033 Marburg, Germany; cookl@staff.uni-marburg.de (L.C.); sengelma@students.uni-marburg.de (M.S.); Winkler8@students.uni-marburg.de (B.W.); conni.nagl93@googlemail.com (C.N.); sarah.koch94@yahoo.de (S.K.); uweschlomann@hotmail.com (U.S.); 2 Department of Visceral Surgery, Philipps University Marburg, Baldingerstr, 35033 Marburg, Germany; slater@med.uni-marburg.de; 3 Center for Systems Biology, Massachusetts General Hospital, 185 Cambridge Street, Boston, MA 02114, USA; Miles.Miller@mgh.harvard.edu; 4 Department of Medicine, Institute for Tumor Immunology, Philipps University Marburg, 35043 Marburg, Germany; poggevon@staff.uni-marburg.de (E.P.v.S.); bastian_doersam@gmx.de (B.D.); preusserc@staff.uni-marburg.de (C.P.)

**Keywords:** tumor microenvironment, extracellular vesicles, ADAM8, lipocalin 2, MMP-9, regulation, PDAC

## Abstract

The metalloprotease-disintegrin ADAM8 is critically involved in the progression of pancreatic cancer. Under malignant conditions, ADAM8 is highly expressed and could play an important role in cell–cell communication as expression has been observed in tumor and immune cells of the tumor microenvironment (TME) such as macrophages. To analyze the potential role of ADAM8 in the TME, ADAM8 knockout PDAC tumor cells were generated, and their release of extracellular vesicles (EVs) was analyzed. In EVs, ADAM8 is present as an active protease and associated with lipocalin 2 (LCN2) and matrix metalloprotease 9 (MMP-9) in an ADAM8-dependent manner, as ADAM8 KO cells show a lower abundance of LCN2 and MMP-9. Sorting of ADAM8 occurs independent of TSG101, even though ADAM8 contains the recognition motif PTAP for the ESCRTI protein TSG101 within the cytoplasmic domain (CD). When tumor cells were co-cultured with macrophages (THP-1 cells), expression of LCN2 and MMP-9 in ADAM8 KO cells was induced, suggesting that macrophage signaling can overcome ADAM8-dependent intracellular signaling in PDAC cells. In co-culture with macrophages, regulation of MMP-9 is independent of the M1/M2 polarization state, whereas LCN2 expression is preferentially affected by M1-like macrophages. From these data, we conclude that ADAM8 has a systemic effect in the tumor microenvironment, and its expression in distinct cell types has to be considered for ADAM8 targeting in tumors.

## 1. Introduction

Extracellular proteolysis is a major process in tumor biology, thereby regulating the proliferation and invasion of tumor cells into the surrounding tissue, and recruitment of immune cells to the tumor site, eventually shaping a tumor-promoting tumor microenvironment [1]. In recent studies, A disintegrin and metalloprotease 8 (ADAM8) was identified as an extracellular metalloprotease-disintegrin important for tumor progression, invasion, and metastasis in pancreatic ductal adenocarcinoma (PDAC) [2]. PDAC is one of the most lethal solid tumors with a 5-year survival rate of less than 8%. In addition, the incidence of PDAC is on the rise and could become a leading cause of cancer deaths by 2030 [3]. A strong desmoplastic stroma response to tumor growth is a hallmark of PDAC and, at least partially, a cause for the devastating patient prognosis [4]. In particular, the tumor microenvironment (TME) with its inflammatory nature activates several immune cell types and suppresses the immune competence of the TME, suggesting an intense communication between tumor and immune cells through the extracellular matrix (ECM). In this regard, ADAM proteases, as membrane-located shedding enzymes, are capable of creating these intercellular signals by the controlled release of membrane proteins involved in immune modulation. One such ADAM protease is ADAM8, a metalloprotease-disintegrin with a proven record of tumor-supporting effects when expressed in tumor cells, thereby facilitating tumor progression, invasion, and immune cell recruitment [2,5]. Recently, a systematic analysis of tumor-associated immune cells in tumor tissues of PDAC patients revealed the additional expression of ADAM8 in macrophages, neutrophils, and NK cells [6]. ADAM8^+^ immune cells can transmigrate through endothelia and invade the ECM, as demonstrated in several inflammation models (e.g., [7,8]). These findings suggest that ADAM8 could also exert its function in immune cells of the TME. Given the strong endogenous expression of ADAM8 in macrophages, we hypothesized that ADAM8 serves important functions in tumor cell–macrophage interactions. These interactions could be mediated by extracellular vesicles (EVs), a defined type of lipid-enclosed particle ranging in size from 30 to 100 nm. ADAM8 was identified as an EV cargo and is of diagnostic value for the early detection of PDAC lesions [9]. In addition, cargo analyses of ADAM8-positive EVs isolated from PDAC patients revealed diagnostic miRNAs correlated with ADAM8 expression levels, such as miR-451 and miR-720. These miRNAs could play a role in regulating cellular functions in the TME as ADAM8-dependent miRNAs. In the current study, we analyzed EV release from PDAC cells and the cellular interactions between macrophages and PDAC tumor cells in an ADAM8-dependent manner. PDAC cell lines with endogenous expression of ADAM8 or a CRISPR/Cas9 knockout of the *ADAM8* gene were co-cultivated with macrophages, and the resulting changes in gene expression were analyzed.

## 2. Results

### 2.1. CRISPR/Cas 9 Knockout of ADAM8 in Tumor Cell Lines MDA MB-231 and Panc89

**Figure 1:**
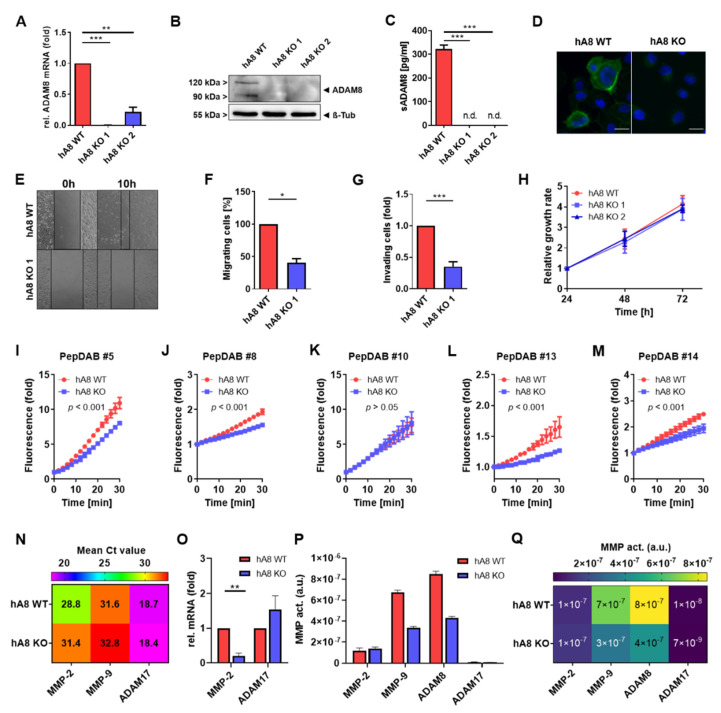
ADAM8 in Panc89 hA8 WT and KO cells. (**A**) mRNA expression, (**B**) Western blot, and (**C**) soluble ADAM8 levels (*n* = 2) in Panc89 hA8 WT and KO 1 and 2. (**D**) Representative immunofluorescence (green) of ADAM8 in Panc89 hA8 WT and KO cells; scale bar, 20 µm. (**A**–**D**) show the successful downregulation of ADAM8 in KO 1 and 2 cells. (**E**,**F**) display scratch assay of Panc89 hA8 WT and KO 1 cells. Images were acquired at 0 h and 10 h (*n* = 2). (**G**) Invasion assay of Panc89 hA8 WT and KO cells in Matrigel using transwell inserts (24 h) demonstrates a decreased invasive behavior in KO cells (*n* = 3). (**H**) Relative growth rates of Panc89 cells show no significant differences between hA8 WT, KO 1, and KO 2 cells (*n* = 2). MMP and ADAM activity assays of Panc89 hA8 WT- and KO cell-derived supernatants (SN) by using PepDAB# (**I**) 5, (**J**) 8, (**K**) 10, (**L**) 13, and (**M**) 14 are illustrated (*n* = 2). (**N**) Heat map of mean Ct values demonstrates the absolute gene expression of MMP-2, MMP-9, and ADAM17, and (**O**) diagram shows the relative mRNA expression of MMP-2 and ADAM17 in Panc89 hA8 WT and KO 1 cells (*n* = 2). (**P**,**Q**) show results of protease activities and cleavage rates of MMP-2, MMP-9, ADAM8, and ADAM17 calculated for hA8 WT and KO 1 cell-derived supernatants by PrAMA inference. Data are presented as mean values ± S.D. * *p* < 0.05, ** *p* < 0.01, *** *p* < 0.001.

Initially, we used cell lines MDA MB-231 and Panc89 for knockout of the *ADAM8* gene, as these cell lines express high endogenous ADAM8 levels. After transfection and selection with puromycin, several representative cell clones were raised and analyzed further. As the effect of ADAM8 deficiency in MB-231 cells has been described extensively [10,11], these cells were only included in some experiments. For Panc89 cells, two representative ADAM8 knockout clones were chosen for the analysis of ADAM8-dependent effects (Figure 1). Loss of ADAM8 expression in Panc89 cells was confirmed in control (“WT”) and KO cell clones by qPCR, Western blot, ELISA, and immunocytochemistry (Figure 1A–D). ADAM8 mRNA was hardly detectable in KO clones by qPCR, and no protein expression was measurable, even by the ELISA assay (Figure 1), where the sensitivity of the assay has a detection limit of 62 pg/mL.

Furthermore, migration was analyzed and showed a reduction for KO 1 (Figure 1E,F). More clones were tested for migration and showed similar results (Supplementary Figure S1A). Proliferation was not affected when comparing control with KO 1 cells (Figure 1H), whereas invasion was significantly reduced upon ADAM8 deficiency (Figure 1G), suggesting that, similar to previous results, ADAM8 has a strong effect on cellular motility in tumor cells. Additionally, another PDAC cell line called AsPC1 was used for CRISPR/Cas9 ADAM8 KO, and the results of AsPC1 and MB-231 ± ADAM8 applied to the here described experiments are shown in Supplementary Figure S2.

To further analyze the potential effect of ADAM8 deficiency on extracellular proteolysis, peptide cleavage assays using five distinct FRET-based peptides were utilized [12], allowing a comparative inference for the proteases MMP-2, MMP-9, ADAM8, and ADAM17 (Figure 1P,Q). Significant ADAM17 activity was not detectable in Panc89 control and KO cells, whereas ADAM17 mRNA expression was detectable with Ct values of ~18 (Figure 1N,O) for both Panc89 control and KO cells. Activity levels of MMP-2 were low with no difference between Panc89 control and KO cells, but mRNA expression of MMP-2 was significantly downregulated in Panc89 KO cells (Figure 1N,O).

In contrast, for MMP-9 and ADAM8, activity levels were reduced in Panc89 KO cells compared to control cells, suggesting that ADAM8 and MMP-9 activities are associated, even though there are very low levels of MMP-9 mRNA (Figure 1N) and protein (Supplementary Figure S1A) detectable in Panc89 cells.

### 2.2. ADAM8 Regulates Intracellular and Extracellular LCN2 Levels in Panc89 Cells

**Figure 2:**
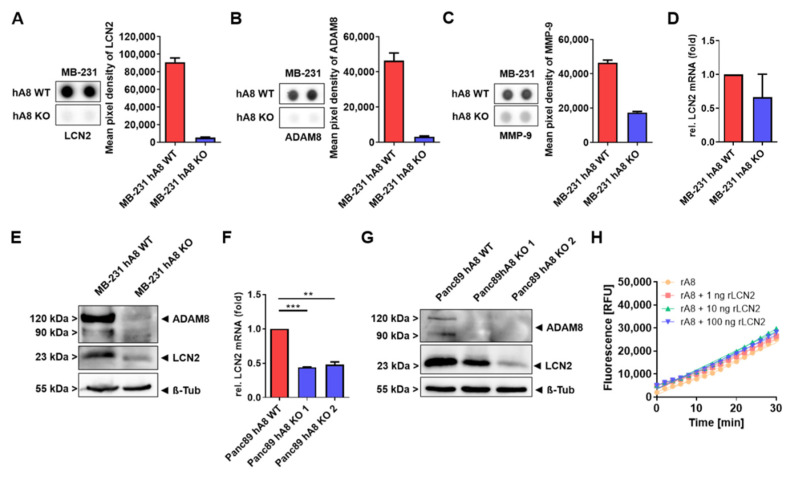
ADAM8 regulates LCN2 levels in tumor cell lines MB-231 and Panc89. The Human Protease/Protease Inhibitory Array demonstrates downregulation of ADAM8 (**A**), MMP-9 (**B**), and LCN2 (**C**) in supernatants derived from MB-231 hA8 KO cells. (**D**) mRNA expression and (**E**) representative Western blot of LCN2 in MB-231 hA8 WT and hA8 KO cells confirm results from (**C**) (*n* = 2). (**F**) mRNA expression and (**G**) representative Western blot of LCN2 in Panc89 hA8 WT, KO 1, and KO 2 cells demonstrate decreased LCN2 expression in hA8 KO cells (*n* = 2). Data are presented as mean values ± S.D., ** p < 0.01, *** p < 0.001. (**H**) Recombinant LCN2 (1 ng, 10 ng, 100 ng) does not affect protease activity of recombinant ADAM8 using a CD23 substrate (PepDAB# 13).

The data shown in Figure 1 suggest that the presence of ADAM8 regulates extracellular proteolytic activity. Thus, we attempted to identify the impact of ADAM8 on the extracellular abundance of other proteases such as MMP family members and potential inhibitors and modulators of protease activity by an antibody-based array screen using MB-231 cells deficient in *ADAM8* (Supplementary Figures S3 and S4). Notably, the strongest effects of the ADAM8 knockout were observed, in addition to ADAM8 itself, for MMP-9 and lipocalin 2 (LCN2, Figure 2A–C). mRNA expression levels of LCN2 were quantified in MB-231 cells by qPCR, showing that the observed downregulation of LCN2 occurs at the protein and, less pronounced, at the mRNA level (Figure 2D). The results obtained for MB-231 cells were confirmed for Panc89 cells (Figure 2F,G), demonstrating decreased relative LCN2 mRNA (Figure 2F). In accordance, the protein expression of LCN2 was decreased in Panc89 hA8 KO cell clones 1 and 2 compared to control cells. More Panc89 hA8 KO clones that were tested for LCN2 at the protein level showed a decrease in protein expression (Supplementary Figure S1B). From these results, we conclude that ADAM8 regulates levels of MMP-9 in MB-231 cells and LCN2 in MB-231 and Panc89 cells. The correlation between ADAM8 and LCN2 levels raised the hypothesis that LCN2 might regulate ADAM8 activity itself. To address this, we performed a peptide cleavage assay using the peptide PepDAB#13 in conjunction with recombinant ADAM8 (50 ng) and recombinant LCN2 at concentrations of 1, 10, and 100 ng and determined ADAM8 activity (Figure 2H). After pre-incubation with recombinant LCN2 for 30 min, we did not find that ADAM8 activity in vitro is affected by LCN2, suggesting that lipocalin 2 is not a physiological inhibitor of ADAM8 activity.

### 2.3. ADAM8 Is Present in EVs as an Active Protease

**Figure 3:**
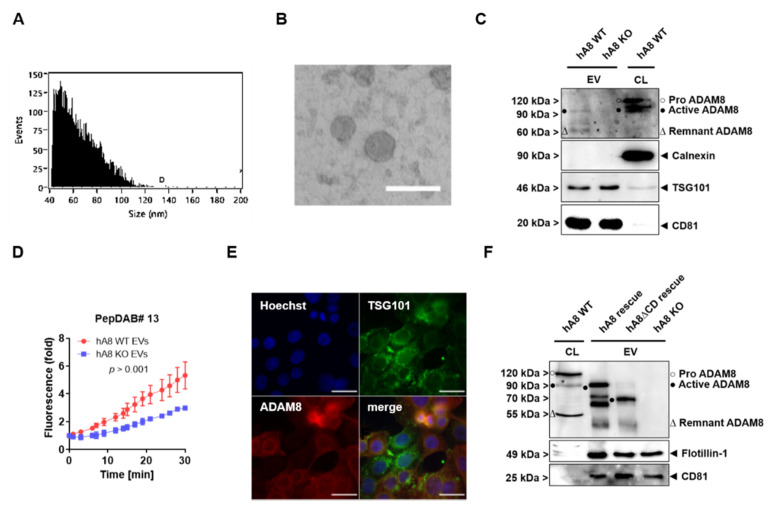
ADAM8 is secreted by Panc89 hA8 WT-derived extracellular vesicles (EV). (**A**) The histogram shows the particle size distribution of EVs isolated from Panc89 cells (analyzed by NanoFCM). (**B**) Electron microscopy of Panc89 hA8 WT-derived EVs demonstrates the successful isolation of EVs; scale bar, 100 nm. Representative Western blot of EVs derived either from Panc89 hA8 WT or KO cells, and cell lysate (CL) of Panc89 hA8 WT cells is shown in (**C**). ADAM8 can be detected as active and remnant ADAM8 in EVs. The negative control Calnexin was not detectable in isolated EVs. The measured activity of Panc89 hA8 WT- and KO-derived EVs on PepDAB #13 is displayed in (**D**) and is upregulated in Panc89 hA8 WT-derived EVs (*n* = 2). (**E**) Representative images of immunofluorescence staining of Panc89 hA8 rescue cells with Hoechst dye (upper left), TSG101 (green; upper right), and ADAM8 (red; lower left). Merged images are displayed in the lower right and show that TSG101 shows little or no co-localization with ADAM8. Scale bar, 50 μm. (**F**) shows the detection of ADAM8, Flotillin-1, and CD81 via Western blot of Panc89 hA8 WT CL and EV preparations isolated from Panc89 hA8 WT, Panc89 hA8 rescue, Panc89 hA8 ΔCD rescue, and Panc89 hA8 KO cells. ADAM8 is detectable in all EV preparations except in EVs isolated from hA8 KO cells. Data are presented as mean values ± S.D.

We further analyzed a possible localization of ADAM8 in extracellular vesicles (EVs). EVs were isolated according to a protocol recommended by the German Society for Extracellular Vesicles (GSEV [13]), using ultracentrifugation and subsequent validation of EV preparations (Figure 3). At first, successful isolation of EVs from Panc89 cells was confirmed by NanoFCM (Figure 3A) and revealed particle sizes of 40–120 nm. Furthermore, electron microscopy was employed to visualize EVs with their membrane composition (Figure 3B). Western blots were performed to detect EV markers Flotillin-1, the ESCRT I protein TSG101, and CD81. All proteins were also tested in whole-cell lysates and demonstrated accumulation of these proteins in EVs from control and ADAM8 KO cells (Figure 3C). Next, we detected the presence of ADAM8 in EVs by Western blots (Figure 3C). In whole-cell lysates of Panc89 control cells, ADAM8 expression was detectable with a strong signal for pro-ADAM8 and the active, mature form. When compared to ADAM8 expression in EVs, mainly the mature form and some lower-molecular weight forms were detected, suggesting that the ADAM8 protein is enriched in EVs as an active protease. In agreement, protease activities were determined for EVs isolated from either control or ADAM8 KO cells (Figure 3D). To investigate the sorting of ADAM8 into EVs, we looked at TSG101, a protein that is part of the ESCRT machinery. TSG101 has been described to recognize PS/TAP motifs at the C-terminus of proteins [14], which can be found at the C-terminal end of the ADAM8 amino acid sequence. Therefore, for that, we checked a possible co-localization of TSG101 (green) and ADAM8 (red) in Panc89 cells (Figure 3E). Therefore, Panc89 hA8 KO cells were used to insert stable full-length ADAM8 (“hA8 rescue”) into the AAVS1 locus. Barely any or no co-localization of ADAM8 and TSG101 was evident in the immunofluorescent staining of Panc89 hA8 rescue cells (Figure 3E, lower right). We also wanted to see whether ADAM8 lacking the PTAP motif would still be packaged into EVs. EVs were isolated from Panc89 hA8 rescue (described in Figure 3E), Panc89 hA8 ΔCD rescue (insertion of ADAM8 without the C-terminal domain), and Panc89 hA8 KO cells. The immunodetection in Figure 3F shows a positive signal for ADAM8 in EVs derived from Panc89 hA8 rescue and Panc89 hA8 ΔCD rescue cells.

### 2.4. ADAM8 Protein Content Correlates with LCN2 Concentration in EVs Derived from Panc89 and MB-231 Cells and MMP-9 Concentration in EVs from MB-231 Cells

**Figure 4:**
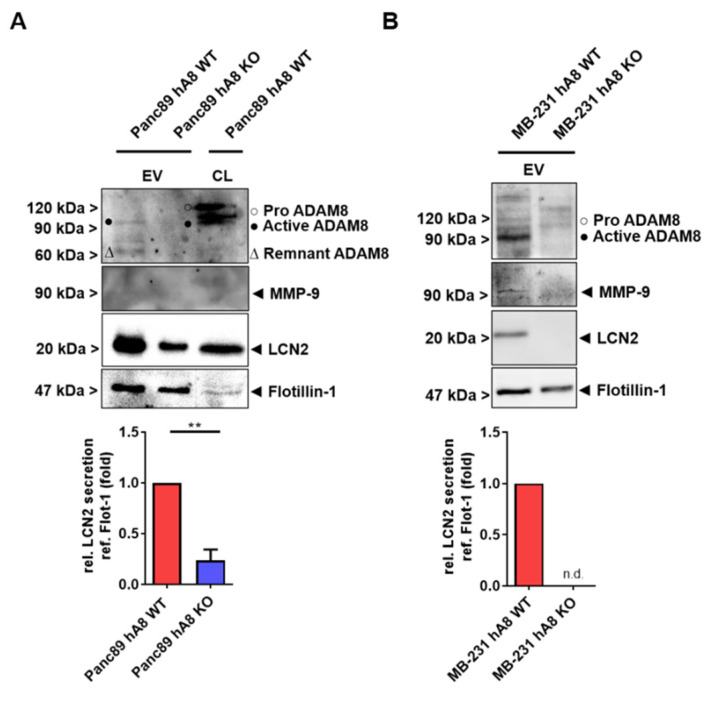
ADAM8 and LCN2 levels correlate in Panc89- and MB-231-derived extracellular vesicles (EV). Representative Western blots (**A**) of EVs derived from either Panc89 hA8 WT or KO cells, and cell lysate (CL) of Panc89 hA8 WT cells, and (**B**) of EVs derived from either MB-231 hA8 WT or MB-231 KO cells show the detection of ADAM8, MMP-9, LCN2, and Flotillin-1 in the upper part. Diagrams below illustrate the quantification and downregulation of LCN2 secretion (relative to Flotillin-1 secretion) in EVs isolated from Panc89 (hA8 WT or hA8 KO 1) or MB-231 (hA8 WT or hA8 KO) cells (*n* = 3). Data are presented as mean values ± S.D. ** *p* < 0.01.

Given the correlation of ADAM8 with the extracellular presence of LCN2 and MMP-9, we next investigated whether EVs containing ADAM8 are composed similarly in Panc89 and MB-231 cells (Figure 4A,B). EVs were isolated from MB-231 and Panc89 tumor cells ± ADAM8, and their protein cargo was analyzed by Western blot. Notably, EVs from ADAM8-deficient MB-231 and Panc89 cells were very low in LCN2. However, in the case of EVs derived from MB-231 cells, the secretion of MMP-9 was lower, whereas in Panc89, MMP-9 was not detectable (Figure 4A).

### 2.5. LCN2 Effect on Downstream Signaling of EGFR in PDAC Cells

**Figure 5:**
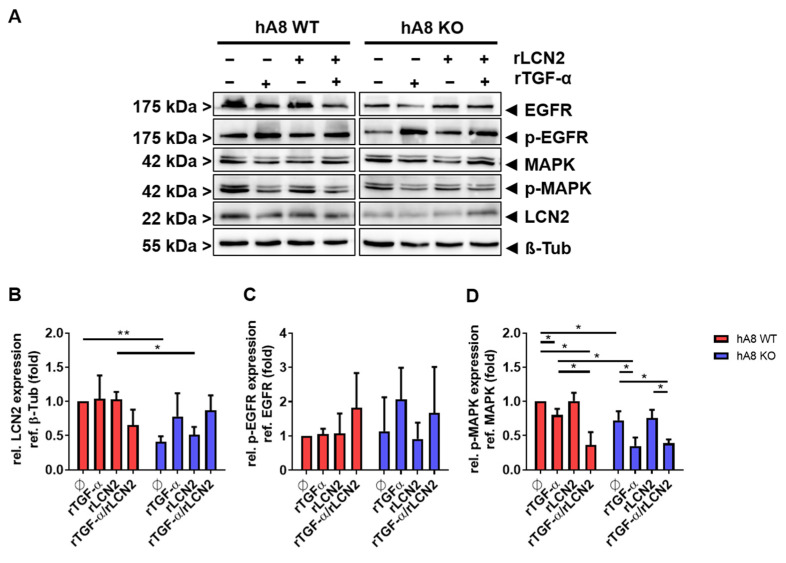
Downstream signaling of EGFR is affected by ADAM8 KO in Panc89 cells. (**A**) Representative immunoblot of EGFR and MAPK phosphorylation, and LCN2 expression after treatment with recombinant LCN2 (rLCN2) and recombinant transforming growth factor-alpha (rTGF-α) for 1 h. (**B**) Quantification of LCN2 shows the downregulation of LCN2 expression in Panc89 hA8 KO cells. The addition of rLCN2 does not increase LCN2 expression in Panc89 hA8 KO cells, whereas rTGF-α alone or combined with rLCN2 adjusts LCN2 expression to Panc89 hA8 WT levels. (**C**) Quantification of p-EGFR illustrates no significant changes in EGFR phosphorylation of Panc89 hA8 KO compared to Panc89 hA8 WT cells. (**D**) The diagram displays the quantification of p-MAPK. The phosphorylation of MAPK is significantly downregulated in Panc89 hA8 KO cells. rTGF-α stimulation decreases MAPK phosphorylation in both Panc89 hA WT and KO cells. Data are presented as mean values ± S.D. * *p* < 0.05, ** *p* < 0.01 (*n* = 2).

The controlled release of LCN2 into the tumor microenvironment could cause effects on EGFR shuttling and on activation of EGFR by phosphorylation, as reported in a recent study [15]. Therefore, we tested the EGFR activation status in Panc89 cells in the absence or presence of ADAM8. In addition, we used exogenous LCN2 in an attempt to compensate for effects caused by downregulation of LCN2 in ADAM8 knockout cells (Figure 5). As a positive control for EGFR activation, TGF-α was used. Figure 5A illustrates the downregulation of LCN2 in Panc89 hA8 KO cells. Apparently, the addition of recombinant LCN2 could not induce endogenous LCN2 expression in KO cells, whereas the application of TGF-α increased LCN2 expression to Panc89 hA8 WT levels (Figure 5B). After quantifying the extent of EGFR activation, no EGFR phosphorylation could be detected in this experiment (Figure 5C). However, lower activation of MAPK could be observed in Panc89 hA8 KO, further enhanced by the addition of TGF-α in WT and KO cells (Figure 5D). Total EGFR and MAPK protein expression was not affected by ADAM8 KO in Panc89 cells (Supplementary Figure S5).

### 2.6. Regulation of LCN2 and MMP-9 in Panc89 Cells after Macrophage Co-Culture

**Figure 6:**
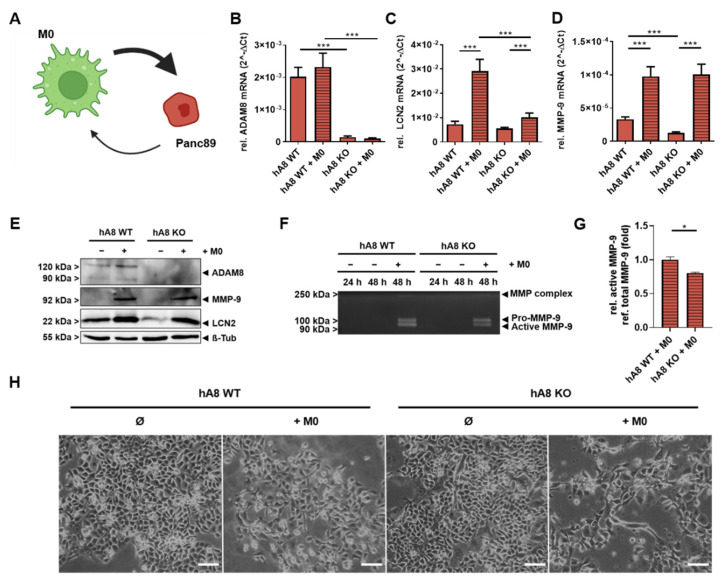
Co-culture of THP1-derived macrophages with Panc89 hA8 WT and KO cells. (**A**) The schematic model depicts the interactions of THP1-derived macrophages (green, M0) and Panc89 cells with or without ADAM8 (red). Created with BioRender.com. (**B**) ADAM8 mRNA expression in both Panc89 hA8 WT and KO is not affected by M0, whereas LCN2 mRNA expression. Data are presented as mean values ± S.D. *** *p* < 0.001. (**C**) is upregulated after co-culture in an ADAM8-dependent manner. Data are presented as mean values ± S.D. *** *p* < 0.001. (**D**) The graph illustrates the upregulation of MMP-9 mRNA expression in both Panc89 hA8 WT and KO after co-culture (*n* = 2). Data are presented as mean values ± S.D. *** *p* < 0.001. (**E**) Representative immunoblot shows the detection of ADAM8, MMP-9, and LCN2 with or without co-culture. In addition to the qPCR results, MMP-9 and LCN2 are upregulated after co-culture at the protein level (*n* = 2). (**F**) Representative zymography of Panc89 hA8 WT and KO cells with or without co-culture demonstrates less active MMP-9 in Panc89 hA8 KO cells than in Panc89 hA8 WT cells after co-culture. (**G**) Quantification of active MMP-9 refers to total MMP-9 in zymography of Panc89 hA8 WT and KO cells after co-culture (*n* = 2). Data are presented as mean values ± S.D. * *p* < 0.05. Representative images of Panc89 cells before and after co-culture are shown in (**H**); scale bar, 100 μm. After co-culture, morphological changes are visible in both Panc89 hA8 WT and KO cells.

An earlier report found a correlation between ADAM8 and MMP-9 when PDAC cells were co-cultivated with macrophages [16]. We investigated this further by addressing whether this effect is ADAM8-dependent and if LCN2 expression is also affected by co-culture with macrophages (Figure 6). In a pre-experiment, we checked ADAM8 mRNA expression and the successful differentiation of THP-1 cells into M0 macrophages (CD68 mRNA expression), as well as the subsequent polarization into M1 (CCL2 mRNA expression) and M2 (CD206 mRNA expression) macrophages. ADAM8 mRNA expression was upregulated in M0, M1, and M2 macrophages. CD68 mRNA expression was significantly increased in M0, M1, and M2, CCL2 mRNA expression in M1, and CD206 mRNA expression in M2 macrophages (Supplementary Figure S6). For the actual co-culture procedure, THP-1 cells were differentiated (M0) for 48 h and then co-cultured with Panc89 cells by inserts of 0.4 μm pore size. Effects on the gene expression of ADAM8 and macrophage-specific genes were determined in M0 macrophages 48 h after starting the co-culture with either Panc89 hA8 WT or KO cells and revealed some expression changes (Supplementary Figure S7). For instance, ADAM8 expression was significantly downregulated in macrophages co-cultivated with Panc89 hA8 WT cells. In addition, the relative expression of the macrophage-specific marker CD68 increased in macrophages co-cultivated with Panc89 hA8 WT cells. With regard to macrophage polarization, CCL2 mRNA expression as an M1 marker was upregulated after co-culture with Panc89 cells and was dependent on ADAM8. In contrast, expression of CD206 as an M2 marker was downregulated after co-culture with Panc89 cells and even less in co-cultures with Panc89 KO cells (Supplementary Figure S7).

These results suggest a subtle effect of tumor cell-derived ADAM8 and signaling on macrophage differentiation in accordance with earlier observations that ADAM8 in macrophages does not affect their differentiation in the TME [17].

We next investigated the effect of THP-1 macrophages on Panc89 cells (Figure 6A). In this experimental setting, ADAM8 expression did not change in Panc89 cells after co-culture in both hA8 WT and KO cells (Figure 6B).

Relative LCN2 mRNA expression levels increased in Panc89 hA8 WT cells after co-culture. As a slight increase in LCN2 mRNA expression was observed in Panc89 KO cells, we conclude that regulation of LCN2 in Panc89 cells is dependent on ADAM8, primarily at the protein level (Figure 6C). Notably, the relative MMP-9 mRNA expression levels were significantly higher in Panc89 cells after co-cultivation with M0 and independent of ADAM8 (Figure 6D). In Western blots, we confirmed the induction of LCN2 and MMP-9 in co-cultured Panc89 cells (Figure 6E). It is interesting to note that expression levels of MMP-9 in Panc89 cells were very low and hardly detectable, and they increased significantly when cells were co-cultured with macrophages. MMP-9 activities released from Panc89 cells after co-culture were assessed by gelatin zymography (Figure 6F,G). MMP-9 activities were significantly increased and suggest that Panc89 cells lacking ADAM8 release lower MMP-9 activities in the TME than Panc89 cells expressing ADAM8 since the amount of pro-MMP-9 is similar in both Panc89 cells lines after 48 h, whereas the amount of active MMP-9 is reduced in Panc89 KO cells (Figure 6F,G). We also checked whether MMP-9 activity could be affected by extrinsic LCN2 by adding recombinant LCN2 to either supernatant derived from co-cultured Panc89 cells for zymography or recombinant MMP-9 for PrAMA analysis (Supplementary Figure S8). It could be determined that recombinant LCN2 had no impact on MMP-9 activity in both experiments. Additionally, co-culture experiments were simulated by adding macrophage-derived supernatants (SN) to Panc89 hA8 WT and KO cells. Subsequently, LCN2 and MMP-9 expression was checked at the mRNA and protein levels (Supplementary Figure S9). Whereas, in this setup, LCN2 expression in Panc89 was not affected by treatment with macrophage-derived SNs, MMP-9 expression was upregulated after the addition of SNs and was dependent on ADAM8. Moreover, the morphology of Panc89 cells dependent on the co-culture with macrophages looked distinct from that of individual Panc89 cells and tended to develop a more mesenchymal character. However, we did not find indications for a “classical” EMT activation, as judged by qPCR analyses for the mRNA expression of N-cadherin, E-cadherin, and the transcription factor ZEB-1 (Figure 6H and data not shown). From these experiments, we conclude that MMP-9 is upregulated after co-culture but independent of ADAM8. However, MMP-9 activation is affected by the presence of ADAM8, which might be related to the regulation of LCN2, which is even more upregulated after co-culture in Panc89 hA8 WT cells than in Panc89 hA8 KO cells. Thus, ADAM8 regulates LCN2 levels and could, thereby, indirectly affect MMP-9 activity.

### 2.7. ADAM8-Dependent Regulation of LCN2 and MMP-9 in Panc89 Cells after Macrophage Polarization

**Figure 7:**
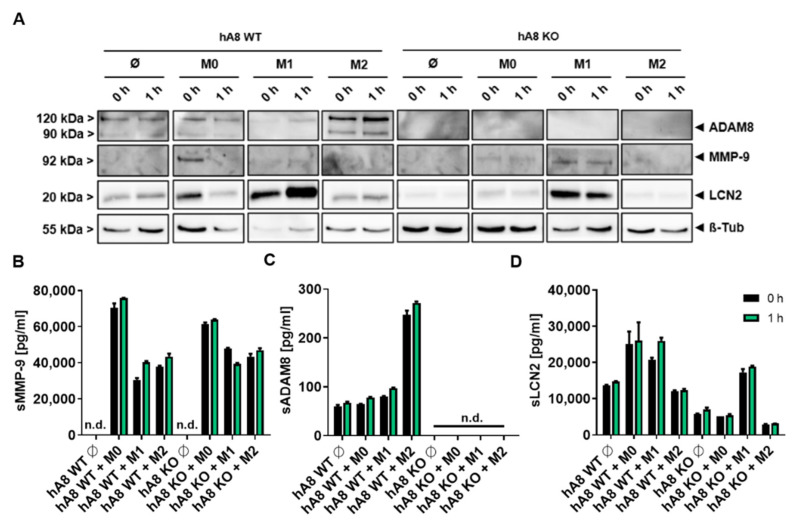
Co-culture of THP1-derived and polarized macrophages with Panc89 hA8 WT and KO cells. (**A**) Western blot illustrates the detection of ADAM8, MMP-9, and LCN2 in Panc89 hA8 WT and KO control cells (Ø) and after co-culture with M0, M1, and M2 macrophages (two time points: 0 h and 1 h). ADAM8 is upregulated in Panc89 hA8 WT cells after co-culture with M2-polarized macrophages. Panc89 cells show the highest MMP-9 expression after co-culture with M0, but M1 macrophages also upregulate MMP-9. LCN2 is dependent on ADAM8 when upregulated in Panc89 cells after co-culture with M0 and M2 macrophages but independent of ADAM8 in Panc89 cells co-cultured with M1 macrophages. (**B**) ADAM8, (**C**) MMP-9, and (**D**) LCN2 ELISA of Panc89 hA8 WT and KO cell-derived supernatants of control cells and after co-culture with M0, M1, and M2 (two time points: 0 h and 1 h). In accordance with the immunoblot results of (**A**), ADAM8 is upregulated in supernatants derived from Panc89 hA8 WT cells after co-culture with M2 macrophages (**B**). At the same time, macrophages increase MMP-9 secretion from an undetectable level to almost 80,000 pg/mL in Panc89 hA8 WT and 60,000 pg/mL in Panc89 hA8 KO cells. M1 and M2 macrophages increase MMP-9 secretion of Panc89 independent of ADAM8, but not as high as in Panc89 cells co-cultured with M0. In contrast, LCN2 is upregulated in Panc89 hA8 WT cells by M0 and M1, but not by M2 macrophages. In the absence of ADAM8, Panc89 hA8 KO cells show low LCN2 secretion in control cells and after co-culture with M0 and M2 macrophages. Only after co-culture with M1 macrophages is the LCN2 secretion level increased (*n* = 1). Data are presented as mean values ± S.D.

Given the strong effect of macrophage co-culture on MMP-9 expression and activity, we next investigated the effect of macrophage polarization on MMP-9 expression in PDAC cells (Figure 7). THP-1 macrophages were differentiated (M0) and polarized (M1, M2) using protocols as previously described [17]. Afterward, co-culture of M0, M1, and M2 macrophages with Panc89 cells was performed for 48 h, and expression levels of ADAM8, LCN2, and MMP-9 in Panc89 cells were determined using Western blot (Figure 7A) and ELISA (Figure 7B–D). Whereas ADAM8 levels in Panc89 cells are strongly induced particularly by M2-polarized macrophages, the increase in MMP-9 levels in Panc89 cells seems not to be dependent on macrophage polarization. For instance, the MMP-9 ELISA results show that Panc89 cells co-cultivated with M0 macrophages had the highest MMP-9 secretion (Figure 7C). For LCN2, Panc89 cells co-cultivated with macrophages of the M1-like polarization type expressed more elevated levels of LCN2 than M2-like macrophages (Figure 7D). Except for co-culture with M1-like macrophages, LCN2 is upregulated in Panc89 cells in an ADAM8-dependent manner. The ADAM8-dependent LCN2 expression in Panc89 cells can be enhanced by co-culture of Panc89 cells with M0 macrophages, as described in Section 2.6.

## 3. Discussion

In this study, we provide insights into the protease-dependent mechanisms underlying the progression of PDAC as a highly invasive tumor entity. First, we demonstrate the essential function of ADAM8 in the extracellular release of MMP-9 and LCN2, two important mediators of cancer progression in PDAC. For instance, a recent report demonstrated that MMP-9, when derived from macrophages, is essential for EMT in tumor cells via activation of protease-activated receptor (PAR-1, [18]). Here, we report on the activation of MMP-9 in tumor cells by macrophages and demonstrate that the communication between macrophages and tumor cells can likely cause MMP-9-dependent effects in an autocrine manner, i.e., by self-induction of MMP-9 in tumor cells when associated with macrophages, as our co-culture experiments suggest. Notably, this effect is not dependent on the polarization state of macrophages, as M1- and M2-like macrophages have similar effects on MMP-9 expression in Panc89 cells. Another strong correlation with ADAM8 expression is reported here for LCN2, a siderophore protein involved in pathogen defense and covalent stabilization of MMP-9. From our data, there are some indications that LCN2 can lead to enhanced activation of pro-MMP-9, thereby causing a higher degree of extracellular proteolysis, leading to the tumor-promoting effect of positive co-regulation of MMP-9 and LCN2 by ADAM8. More directly, LCN2 was recently investigated in a mouse model of PDAC. LCN2-deficient mice were crossed with transgenic mice expressing Kras^G12D^ in acinar cells [19]. In these mice, the lack of LCN2 caused a significant reduction in immune cell infiltration, PanIN, and tumor growth, suggesting that LCN2 in the TME is an important determinant for PDAC progression and patient prognosis, similar to MMP-9. Thus, ADAM8 could amplify its detrimental function in PDAC by simultaneously affecting MMP-9 and LCN-2 expression. Given the finding that LCN2 can determine MMP-9 activity in vivo, it can be hypothesized that MMP-9 activity in the TME is directly affected. However, in contrast to the regulation of MMP-9, LCN2 is differentially regulated by co-culturing PDAC cells with macrophages. Undifferentiated as well as M1-polarized macrophages cause increased LCN2 release, whereas M2-polarized macrophages suppress LCN2 secretion. Thus, a distinct regulation of MMP-9 and LCN2 expression by macrophages could imply that both proteins serve functions distinct from the regulation of net protease activity in the TME. A limitation of this study is the sole use of THP-1 cells rather than primary macrophages derived from PBMCs. To address this issue, we tried to include these cell sources in our analysis; however, we obtained heterogeneous results that prevent a conclusive interpretation. A further limitation is the analysis of 2D co-cultures that might not reflect the interaction of tumor cells with macrophages in vivo. In this regard, a 3D model using organoid co-cultures will be required to get closer to a more authentic tumor microenvironment. Since we could demonstrate that only MMP-9 and not LCN2 expression is affected in Panc89 cells after treatment with M0-derived supernatants, another limitation of this study is the treatment of Panc89 cells with THP-1 macrophage-derived EVs. As EVs can function as a carrier of signaling molecules, we hypothesize that EV treatment could induce LCN2 expression and therefore MMP-9 activity, which is a matter of further studies.

Other important findings in this study are that ADAM8 is sorted into EVs, and the ADAM8 dependence of MMP-9 and LCN2 is found, in addition to lysates, in the EV cargo; thus, we can conclude that ADAM8 is a key regulator of MMP-9 and LCN2 release in the TME. However, the mechanism by which ADAM8 is sorted into EVs remains unclear. Besides the most described ESCRT machinery, many ESCRT-independent cargo and biomolecule sorting pathways in EVs have been reported [20]. TSG101, as part of the ESCRT complex, can recognize the tetrapeptide protein motif PS/TAP [14]. The amino acid motif PTAP is found in ADAM8 at the very C-terminus (aa residues 821–824). To demonstrate the function of this motif, we established a Panc89 cell line lacking the C-terminal domain of ADAM8, thereby deleting the PTAP motif of ADAM8, and expected this form of ADAM8 not to be present in EVs. Unexpectedly, the C-terminal mutant form of ADAM8 is still capable of being secreted in EVs. Furthermore, the lack of co-localization of TSG101 with ADAM8 supports the hypothesis that the internalization of ADAM8 in EVs occurs independently of the ESCRT-I protein TSG101. ESCRT-independent pathways such as lipid raft-, tetraspanin-, and ceramide-mediated mechanisms could exhibit potential ADAM8 EV cargo loading alternatives that must be investigated [21]. Tan et al. [22] demonstrated that lipid rafts could conduce to a platform for exosomal biogenesis in mesenchymal stem cells. ADAM17 has been described as an active protease associated with lipid rafts [23] and could indicate a possible mechanism for ADAM17 EV encapsulation, and thus for ADAM8. In addition, the release of ADAM10 in EVs by melanoma cells was shown to be linked to a paxillin/integrin interaction and a subsequent shift to lipid rafts [24], supporting the suggested mechanism for ADAM8 exosomal release. In EVs, ADAM8 can be detected as an active protease, as peptide cleavage assays with WT and KO cells demonstrate. Thus, the topological orientation of ADAM8 in EVs is likely to be directed to the extracellular compartment where ADAM8-dependent cleavage can occur. In some reports, ADAM8 was also discussed as a membrane protein mediating cell–cell fusion, where ADAM8 on the surface of EVs could potentially contribute to the fusion of EVs with adjacent cells to release their cargo [25]. In addition, as ADAM8 can regulate miRNA expression in tumor cells (Schäfer et al., manuscript submitted), it is likely that ADAM8–ADAM8 interactions between EVs and ADAM8-expressing cells could lead to fusion events and facilitate the transfer of miRNAs into cells to regulate gene expression. In the case of ADAM8-positive EVs in PDAC, such miRNAs were reported to be miRNAs 720 and 451 [9]. Concomitant with the ADAM8 content in EVs, MMP-9 and LCN2 are affected, suggesting that ADAM8 regulates the amount of these proteins in the TME. A cell-autonomous effect of ADAM8 in tumor cells was extensively described in numerous studies. However, the function of ADAM8 in the TME, given the localization in EVs, is worth further studies involving other immune cell types such as neutrophils and natural killer cells in which ADAM8 is also highly expressed.

## 4. Materials and Methods

### 4.1. Cell Culture

Panc89 cells were kindly provided by Prof. Anna Trauzold, Kiel University, and were cultivated in RPMI (Gibco^TM^, Life Technologies, Carlsbad, CA, USA) supplemented with 10% (*v*/*v*) Fetal Bovine Serum (FBS; Sigma-Aldrich, Munich, Germany), 0.1 mg/mL Penicillin-Streptomycin (Gibco^TM^, Life Technologies, Carlsbad, CA, USA), and 1 mM Sodium Pyruvate (Gibco^TM^, Life Technologies, Carlsbad, CA, USA) in a humidified atmosphere at 37 °C and 5% CO_2_. MB-231 cells were purchased from American Type Culture Collection (ATCC, Manassas, VA, USA) and were cultivated in DMEM (Gibco^TM^, Life Technologies, Carlsbad, CA, USA) with the same additives as described for Panc89 cells.

### 4.2. Generation of Stable Panc89 and MB-231 ADAM8 Knockout Cells

To generate a genomic ADAM8 knockout in Panc89 and MB-231 cells, the ADAM8 Human Gene Knockout Kit (CRISPR) from OriGene (CAT#: KN213386, Rockville, ML, USA) was used. The transfection of the cells and further necessary steps were performed as previously described [11]. Single-cell clones were obtained after the selection with 1 μg/mL Puromycin (InvivoGen, San Diego, CA, USA).

### 4.3. Generation of Stable Panc89 ADAM8 Rescue Cells

For a genomic knock-in of the full-length ADAM8 or an ADAM8 lacking the C-terminal domain in Panc89 hA8 KO cells, the ADAM8 AAVS1 Transgene Kockin kit (BSD) from OriGene (CAT#: GE100036, Rockville, ML, USA) was used. The transfection of the cells and further necessary steps were performed as described in Section 4.2. Single-cell clones were obtained after the selection with 10 μg/mL Blasticidin (InvivoGen, San Diego, CA, USA).

### 4.4. THP-1 Cell Differentiation and Polarization

For macrophage differentiation, THP-1 cells were seeded at a density of 500,000 cells per well of a 6-well plate and treated with 10 ng/mL phorbol 12-myristate 13-acetate (PMA, Sigma-Aldrich, Munich, Germany). After 48 h, differentiated THP-1 cells were exposed for another 6 h to either 50 ng/mL LPS (Sigma-Aldrich, Munich, Germany) and 20 ng/mL IFN-γ (PeproTech, Hamburg, Germany) for M1 polarization or 20 ng/mL IL-4 (PeproTech, Hamburg, Germany) for M2 polarization.

### 4.5. Co-Culture of Panc89 with THP-1

At a density of 500,000 cells per approach, THP-1 cells were seeded in the upper part of ThinCert^TM^ Cell Culture Insert with a 0.4 µm diameter (Greiner Bio-One GmbH, Frickenhausen, Germany). After 48 h of differentiation, 500,000 Panc89 cells were seeded in the lower compartment (well) and left in co-culture for another 48 h. Cells were either harvested for RNA isolation or protein extraction and subsequent Western blot analysis. For M1 and M2 co-culture, THP-1 cells were treated with 50 ng/mL LPS (Sigma-Aldrich, Munich, Germany) and 20 ng/mL IFN-γ (PeproTech, Hamburg, Germany) for M1 polarization or 20 ng/mL IL-4 (PeproTech, Hamburg, Germany) for M2 polarization 6 h after differentiation and before co-culture. The described co-culture procedure was performed considering different established protocols, as described, for example, in Landmann and Buchholz, Department of Gastroenterology, Endocrinology, Metabolism, and Infectiology, Philipps University Marburg https://archiv.ub.uni-marburg.de/ubfind/Record/urn:nbn:de:hebis:04-z2019-0310 (accessed on 29 December 2021) and [26,27].

### 4.6. EV Isolation and Characterization by NanoFCM and Electron Microscopy

After the tumor cells reached 80% confluency, the medium was changed to RPMI medium without Phenol Red (Gibco^TM^, Life Technologies, Carlsbad, CA, USA) but supplemented with 1% (*v*/*v*) Insulin-Transferrin-Selenium (Gibco^TM^, Life Technologies, Carlsbad, CA, USA). After 72 h, the supernatants were centrifuged at 300× *g* for 10 min followed by an additional centrifugation step at 3000× *g* for 10 min. The resulting supernatant was filtered (0.2 μm) and subsequently centrifuged at 10,000× *g* for 1 h at 4 °C. EVs were pelleted at 100,000× *g* for 90 min at 4 °C in an Optima XPN-80 ultracentrifuge (Beckman Coulter, Krefeld, Germany) with an SW32Ti swing-out rotor, resuspended in 700 μm HBSS (Gibco^TM^, Life Technologies, Carlsbad, CA, USA), and again centrifuged at 100,000× *g* for 90 min at 4 °C in an Optima MAX-XP (Beckman Coulter, Krefeld, Germany) ultracentrifuge with a TLA-55 fixed angle rotor. Finally, the EVs were resuspended in 50 μL HBSS (Gibco™, Life Technologies, Carlsbad, CA, USA). For particle size and quantity determination, EVs were applied to a flow nano analyzer (NanoFCM Co. Ltd., Nottingham, UK). Electron microscopy was conducted as previously described [9].

### 4.7. RNA Isolation and Quantitative Real-Time PCR

For total RNA isolation, cells were resuspended in 1 mL Qiazol (Qiagen, Hilden, Germany) and 200 μL Chloroform was added. After mixing the samples, the lysed cells were incubated at RT for 5 min and subsequently centrifuged at 12,000× *g* for 15 min at 4 °C. The RNA in the upper aqueous phase was then precipitated by adding 500 μL Isopropanol. After repeated centrifugation, the RNA was washed with 75% (*v*/*v*) Ethanol, dried, and dissolved in 10–30 μL RNase-Free Water. The resulting concentration of the isolated RNA was determined by NanoPhotometer^®^ NP80 (Implen, Munich, Germany). An amount of 2 µg of isolated RNA was applied to RNA to cDNA EcoDry^TM^ kit (Takara Bio Inc., Kusatsu, Japan) for reverse transcription. The procedure was conducted according to the manufacturer’s instructions. Quantitative real-time PCR was performed using iTaq^TM^ Universal SYBR^®^ Green Supermix (Bio-Rad Laboratories GmbH, Feldkirchen, Germany), cDNA equivalent of 20 ng total RNA, and either forward and reverse primers or QuantiTect Primer Assay (Qiagen, Hilden, Germany) in a 20 µL total PCR reaction volume in a StepOnePlusTM Real-Time PCR System (Thermo Fisher Scientific, Waltham, MA, USA). Relative changes in mRNA expression were calculated using the ΔCt method using XS13 as a housekeeping gene (the sequence was described elsewhere) [2].

### 4.8. rLCN2 and rTGF-α Stimulation of Panc89 Cells

A total of 500,000 cells were seeded in each well of a 6-well plate. After 48 h, the medium was changed to RPMI (Gibco^TM^, Life Technologies, Carlsbad, CA, USA) Medium supplemented with 0.5% (*v*/*v*) FBS (Sigma-Aldrich, Munich, Germany) and cultivated for another 6 h. Starved cells were then treated with either rTGF-α (PeproTech, Hamburg, Germany), rLCN2 (R&D Systems, Minneapolis, MN, USA), or their combination for 1 h. Stimulated cells were then applied to total protein extraction and Western blot analysis.

### 4.9. Protein Extraction and Western Blot Analysis

Total protein extraction was performed by detaching the cells with a cell scraper. Cells were washed with PBS, resuspended in 50 μL RIPA (50 mM HEPES pH 7.4; 150 mM NaCl; 1% (*v*/*v*) NP-40; 0.5% (*w/v*) Natriumdeoxycholate; 0.1% (*w/v*) SDS; 10 mM Phenantrolin; 10 mM EDTA; Pierce^TM^ Protease Inhibitor Mini Tablets, EDTA-free, Thermo Scientific, Waltham, MA, USA; Pierce^TM^ Phosphatase Inhibitor Mini Tablets, Thermo Scientific, Waltham, MA, USA) and incubated for 30 min on ice. After centrifugation at 12,000× *g* for 5 min at 4 °C, the protein concentration of the supernatant containing the total protein was determined by Pierce^TM^ BCA Protein Assay Kit (Thermo Scientific, Waltham, MA, USA). Proteins from equal amounts of lysate were then boiled in 1× loading buffer (5× loading buffer: 60 mM Tris-HCl pH 6.8; 2% (*w/v*) SDS; 10% (*w/v*) Glycerol; 5% (*v*/*v*) ß-Mercaptoethanol; 0.01% (*w/v*) Bromphenol-Blue) for 5 min. The proteins were separated by 10% SDS-PAGE and subsequently transferred to a nitrocellulose membrane (GE Healthcare, Chicago, IL, USA). The membrane was then blocked with 5% (*w/v*) milk powder (MP) in TBST (50 mM Tris, pH 7.5; 150 mM NaCl; 0.1% (*w/v*) Tween-20) for 1 h and probed with the following primary antibodies: anti-ADAM8 (PA5-47047, Thermo Fisher Scientific, Waltham, MA, USA; 1:1000 in 5% MP in TBST), anti-beta Tubulin (NB600-936, Novus Biological, Littleton, CO, USA; 1:2000), anti-LCN2 (AF1757, R&D Systems, Minneapolis, MN, USA; 1:1000), anti-EGFR (4267, Cell Signaling Technology, Danvers, MA, USA; 1:1000), anti-pEGFR (3777, Cell Signaling Technology, Danvers, MA, USA; 1:1000), anti-MAPK (4696, Cell Signaling Technology, Danvers, MA, USA; 1:2000), anti-pMAPK (4370, Cell Signaling Technology, Danvers, MA, USA; 1:2000), Calnexin (2679, Cell Signaling Technology, Danvers, MA, USA; 1:1000), anti-Flotillin-1 (PA5-18053, Thermo Scientific, Waltham, MA, USA; 1:2000), anti-TSG101 (ab83, Abcam, Cambridge, UK; 1:1000), MMP-9 (AF911, R&D Systems, Minneapolis, MN, USA; 1:2000), and anti-CD81 (sc166029, Santa Cruz Biotechnology, Dallas, TX, USA; 1:500), at 4 °C overnight. After washing 3 times with TBST, the membrane was incubated with horseradish peroxidase-conjugated secondary antibodies for 1 h (Abcam, Cambridge, UK; 1:5000) followed by a repeated washing step. Detection was performed by using Chemiluminescent HRP Substrate, Western Bright Sirius (Advansta Inc., San Jose, CA, USA).

### 4.10. Human Protease/Protease Inhibitor Array

For the Proteome Profiler Human Protease/Protease Inhibitor Array (ARY025, R&D Systems, Minneapolis, MN, USA), 500,000 MB-231 cells were seeded in each well of a 6-well plate overnight. The next day, the medium was changed to Gibco^TM^, Life Technologies, Carlsbad, CA, USA) supplemented with 2% (*v*/*v*) FBS (Sigma-Aldrich, Munich, Germany) for 48 h. The supernatants were collected, and protein concentration was determined by NanoPhotometer^®^ NP80 (Implen, Munich, Germany). Concentrations were adjusted, and the array was carried out according to the manufacturer’s protocol.

### 4.11. Immunofluorescence Staining

Before the procedure, coverslips were coated with 50 µg/mL Collagen Type I from rat tail (Sigma-Aldrich, Munich, Germany) and incubated for 1 h at 37 °C. The coating was then washed 2 times with PBS, and subsequently, Panc89 cells were seeded at a density of 100,000 cells per 0.5 mL on the coated coverslips. After 24 h, cells were washed 3 times with PBS and then fixed with 4% (*w/v*) PFA for 15 min followed by 1 h blocking with 5% (*w/v*) BSA in PBS. Primary ADAM8 (PA5-47047, Thermo Fisher Scientific, Waltham, MA, USA; 1:100) or TSG101 (ab83, Abcam, Cambridge, UK; 1:100) antibody was added for overnight incubation at 4 °C. The next day, cells were washed 3 times with PBS and incubated with secondary antibody Alexa Fluor 488 (A-11055, Thermo Fisher Scientific, Waltham, MA, USA; 1:500), Alexa Fluor 488 (ab150105, Abcam, Cambridge, UK; 1:500), or Texas Red (PA1-28662, Thermo Fisher Scientific, Waltham, MA, USA; 1:1000) for 1 h at RT. After another washing step, cells were incubated with Hoechst 33,342 dye (Sigma-Aldrich, Munich, Germany) for counterstaining for 20 min. Images were obtained using a Leica DM 5500 microscope (Leica Microsystems, Wetzlar, Germany).

### 4.12. Gelatin Zymography

After 48 h of co-culture, cells were cultivated in monoculture for another 24 h with 1 mL RPMI (Gibco^TM^, Life Technologies, Carlsbad, CA, USA) without phenol red and FBS, but supplemented with 1% (*v*/*v*) Insulin-Transferrin-Selenium (Gibco^TM^, Life Technologies, Carlsbad, CA, USA). The supernatants were collected, centrifuged at 4000× *g* for 10 min, and concentrated with Vivaspin (Sartorius AG, Göttingen, Germany). Protein concentration determination was performed as described above. Before the electrophoresis, the samples were either treated with 0.6 mM APMA (Sigma-Aldrich, Munich, Germany), with recombinant LCN2 (R&D Systems, Minneapolis, MN, USA), or with both for 1 h at 37 °C. The samples were then diluted with 2x non-reducing loading dye (1 M Tris-HCl, pH 6.8; 20% (*w/v*) Glycerol; 10% SDS (*w/v*); 0.1% (*w/v*) Bromphenol-Blue) and loaded on a gel containing 0.1% (*w/v*) Gelatin in the separating gel. After the run, the gel was initially washed 2 times with renaturation buffer (2.5% (*v*/*v*) Triton-X-100) for 30 min each, followed by equilibration with developing buffer (1 M Tris-HCl, pH 7.5; 200 mM NaCl; 4 mM CaCl_2_; 0.02% (*v*/*v*) Brij-35) for 30 min and subsequent incubation in a fresh developing buffer for 16 h at 37 °C. The next day, the gel was stained with Coomassie staining solution (50% (*v*/*v*) Methanol, 10% (*v*/*v*) Acetic acid, 0.5% (*w/v*) Coomassie Brilliant Blue G250) for 1 h and destained with destaining solution (50% (*v*/*v*) Methanol, 10% (*v*/*v*) Acetic acid) until the bands of the active enzyme were visible.

### 4.13. Protease Activity Assay in Panc89-Derived Supernatants or EVs

Both supernatants and EVs derived from Panc89 cells were tested for ADAM and MMP activities by determining the cleavage of FRET-based polypeptide substrates with a high Kcat/Km for different proteases (PEPDab5, PEPDab8, PEPDab10, PEPDab13, PEPDab14, BioZyme, Inc., Apex, NC, USA), as previously described [28]. For the conditioned supernatants, 500,000 cells were seeded in each well of a 6-well plate. After 24 h, the medium was changed to 1 mL RPMI (Gibco^TM^, Life Technologies, Carlsbad, CA, USA)without phenol red and FBS, but supplemented with 1% (*v*/*v*) Insulin-Transferrin-Selenium (Gibco^TM^, Life Technologies, Carlsbad, CA, USA) for another 24 h. After the incubation, the supernatants were centrifuged at 4000× *g* for 10 min. Briefly, 10 µM of each PEPDab in 50 µL assay buffer (1 mM ZnCl2, 20 mM Tris-HCl pH 8.0, 10 mM CaCl2, 150 mM NaCl, 0.0006% Brij-35) was incubated with either 50 µL conditioned supernatant or 5 × 10^9^ EVs in a total volume of 100 µL. The resulting fluorescence was monitored every 2 min for 6 h at 37 °C with a multiwell plate reader (FLUOstar OPTIMA, BMG Labtech, Offenburg, Germany) using a λex of 485 nm and a λem of 530 nm.

### 4.14. ELISA

Soluble ADAM8 (DY1031, R&D Systems, Minneapolis, MN, USA), LCN2 (DY1737, R&D Systems, Minneapolis, MN, USA), and MMP-9 (DY911, R&D Systems, Minneapolis, MN, USA) levels in Panc89-derived supernatants were determined by using DuoSet ELISA kits. A total of 500,000 cells were incubated in a normal growth medium (as described above) in each well of a 6-well plate for 48 h. Before the procedure, the collected supernatants were centrifuged at 4000× *g* for 10 min and subjected to ELISA according to the manufacturer’s protocol.

### 4.15. Proliferation Assay

A total of 5000 cells per well were seeded in triplicates in a 96-well plate. After 24 h, 48 h, and 72 h, viability was determined by adding 50 µL CellTiter-Glo^®^ 3D Cell Viability Assay (Promega, Walldorf, Germany), followed by a shaking step for 15 min and subsequent incubation for 15 min at RT in the dark. The resulting luminescence was measured using a multiwell plate reader (FLUOstar OPTIMA, BMG Labtech, Offenburg, Germany).

### 4.16. Scratch Assay

The scratch assay was performed by seeding 30,000 cells per well of a Culture-Insert 2 Well (ibidi GmbH, Gräfelfing, Germany) inserted in a 24-well plate. After 6 h and allowing the cells to attach, the normal growth medium was changed to RPMI Medium (Gibco^TM^, Life Technologies, Carlsbad, CA, USA) supplemented with 0.5% (*v*/*v*) FBS (Sigma-Aldrich, Munich, Germany) for overnight starvation. The Culture-Insert 2 Well was removed, and cells were washed with a fresh normal growth medium to remove non-adherent cells. From this moment, images at each edge of the gap were taken at time points 0 h and 10 h. The images were analyzed using the Image J software plugin “Wound_ healing_size_tool_updated” published by [29].

### 4.17. Invasion Assay

To examine the cells’ invasive behavior, an invasion assay was performed using ThinCert^TM^ Cell Culture Inserts with an 8 µm pore diameter (Greiner Bio-One GmbH, Frickenhausen, Germany). Before seeding the cells, the upper side of the Thincert was coated with 50 µL Basement Membrane Matrix Growth Factor Reduced Matrigel (Corning^®^, Corning, NY, USA). After 1 h incubation at 37 °C, 25,000 cells were seeded in 50 µL RPMI Medium (Gibco^TM^, Life Technologies, Carlsbad, CA, USA) supplemented with 0.5% (*v*/*v*) FBS (Sigma-Aldrich, Munich, Germany) at the lower side of the Thincert. Cells were allowed to attach to the membrane for a maximum of 4 h. Subsequently, 250 µL RPMI Medium (Gibco^TM^, Life Technologies, Carlsbad, CA, USA) supplemented with 20% (*v*/*v*) FBS (Sigma-Aldrich, Munich, Germany) was added to the upper part of the Thincert and 750 µL RPMI Medium (Gibco^TM^, Life Technologies, Carlsbad, CA, USA) supplemented with 0.5% (*v*/*v*) FBS (Sigma-Aldrich, Munich, Germany) to the well. Cells were then allowed to invade the Matrigel due to an FBS gradient. After 24 h, the Thincerts were treated with 4% (*w/v*) PFA for 30 min, and subsequently, cells were permeabilized with 0.3% (*v*/*v*) Triton-X for another 30 min. For counting and quantification, cells were stained with Hoechst 33,342 (Thermo Scientific, Waltham, MA, USA) dye overnight. Z-Stacks of five random viewing fields were recorded, and cells on the lower part of the Thincert (non-invasive) and cells in the matrigel (invasive) were counted.

### 4.18. Statistical Analysis

Student’s *t*-test or two-way ANOVA was applied for statistical analysis. Data were considered not significant (*p* > 0.05, no asterisk), significant * (*p* < 0.05), highly significant ** (*p* < 0.01), or very highly significant *** (*p* < 0.001) and are expressed as the mean ± S.D.

## 5. Conclusions

Taken together, our study sheds more light on the role of ADAM8 in the TME by providing evidence that effects observed for ADAM8 in a cell-autonomous manner can be overcome by co-culture with macrophages in a “systemic” mode, i.e., by intense communication between tumor and immune cells that could be mediated by EVs. When considering ADAM8 as a potential drug target in PDAC, these findings have to be taken into account.

## Data Availability

All data presented here are available on request to the corresponding author.

## Supplementary Materials

The following supporting information can be downloaded at: https://www.mdpi.com/article/10.3390/ijms23041976/s1.
