# Supplementary material for: ADAM8-Dependent Extracellular Signaling in the Tumor Microenvironment Involves Regulated Release of Lipocalin 2 and MMP-9
Source: Int J Mol Sci. 2022 Feb 10;23(4):1976. doi: 10.3390/ijms23041976 (PMC8875419; doi:10.3390/ijms23041976)
Supplement: Supplementary file 1 [file ijms-23-01976-s001.zip › ijms-1556195-supplementary.pdf]

International Journal of *Molecular Sciences*

MDPI

Supplementary materials

ADAM8-Dependent Extracellular Signaling in the Tumor Microenvironment Involves Regulated Release of Lipocalin 2 and MMP-9

Lena Cook [1], Marie Sengelmann [1], Birte Winkler [1], Constanze Nagl [1], Sarah Koch [1], Uwe Schlomann [1,2], Emily P. Slater [2], Miles A. Miller [3], Elke Pogge von Strandmann [4], Bastian Dörsam [4], Christian Preußer [4] and Jörg W. Bartsch [1,*]

1 Department of Neurosurgery, Philipps University Marburg, Baldingerstr, 35033 Marburg, Germany; cookl@staff.uni-marburg.de (L.C.); sengelma@students.uni-marburg.de (M.S.); Winkler8@students.uni-marburg.de (B.W.); conni.nagl93@googlemail.com (C.N.); sarah.koch94@yahoo.de (S.K.); uweschlomann@hotmail.com (U.S.)

2 Department of Visceral Surgery, Philipps University Marburg, Baldingerstr, 35033 Marburg, Germany; slater@med.uni-marburg.de

3 Center for Systems Biology, Massachusetts General Hospital, 185 Cambridge Street, Boston, MA 02114, USA; Miles.Miller@mgh.harvard.edu

4 Institute for Tumor Immunology, Department of Medicine , Philipps University Marburg, 35043 Marburg, Germany; poggevon@staff.uni-marburg.de (E.P.v.S.); bastian_doersam@gmx.de (B.D.); preusserc@staff.uni-marburg.de (C.P.)

* Correspondence: jbartsch@med.uni-marburg.de; Tel.: +49-6421-58-61173

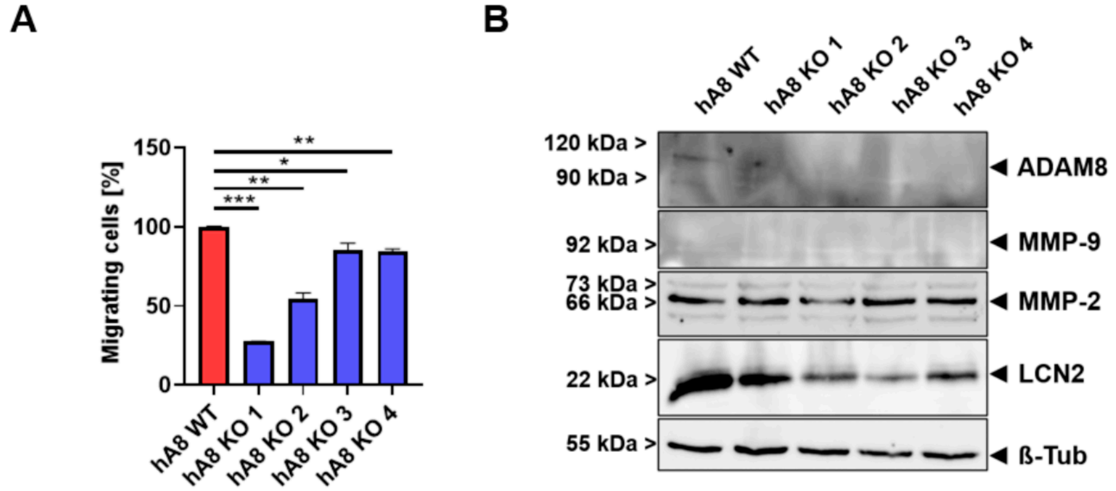

Figure S1. ADAM8 regulates migration and LCN2 expression in Panc89 cells. (**A**) Quantification is displayed of scratch assay performed with Panc89 hA8 WT, and KO 1, 2, 3, 4 cells. Images were acquired at 0 h and 10 h. ($n = 2$). Migration behavior was significantly downregulated in all knockout clones. Data were given as mean values ± S.D. * $p < 0.05$, ** $p < 0.01$, *** $p < 0.001$. (**B**) Representative western blot of ADAM8, MMP-9, MMP-2, LCN2, and s-Tubulin of Panc89 hA8 WT, KO 1, KO 2, KO 3, and KO 4 cells. ADAM8 was only detectable in Panc89 hA8 WT cells. No signal was visible for MMP-9 immunodetection. MMP-2 protein expression was not affected by Panc89 hA8 KO. LCN2 expression was markedly reduced in all Panc89 hA8 KO clones.

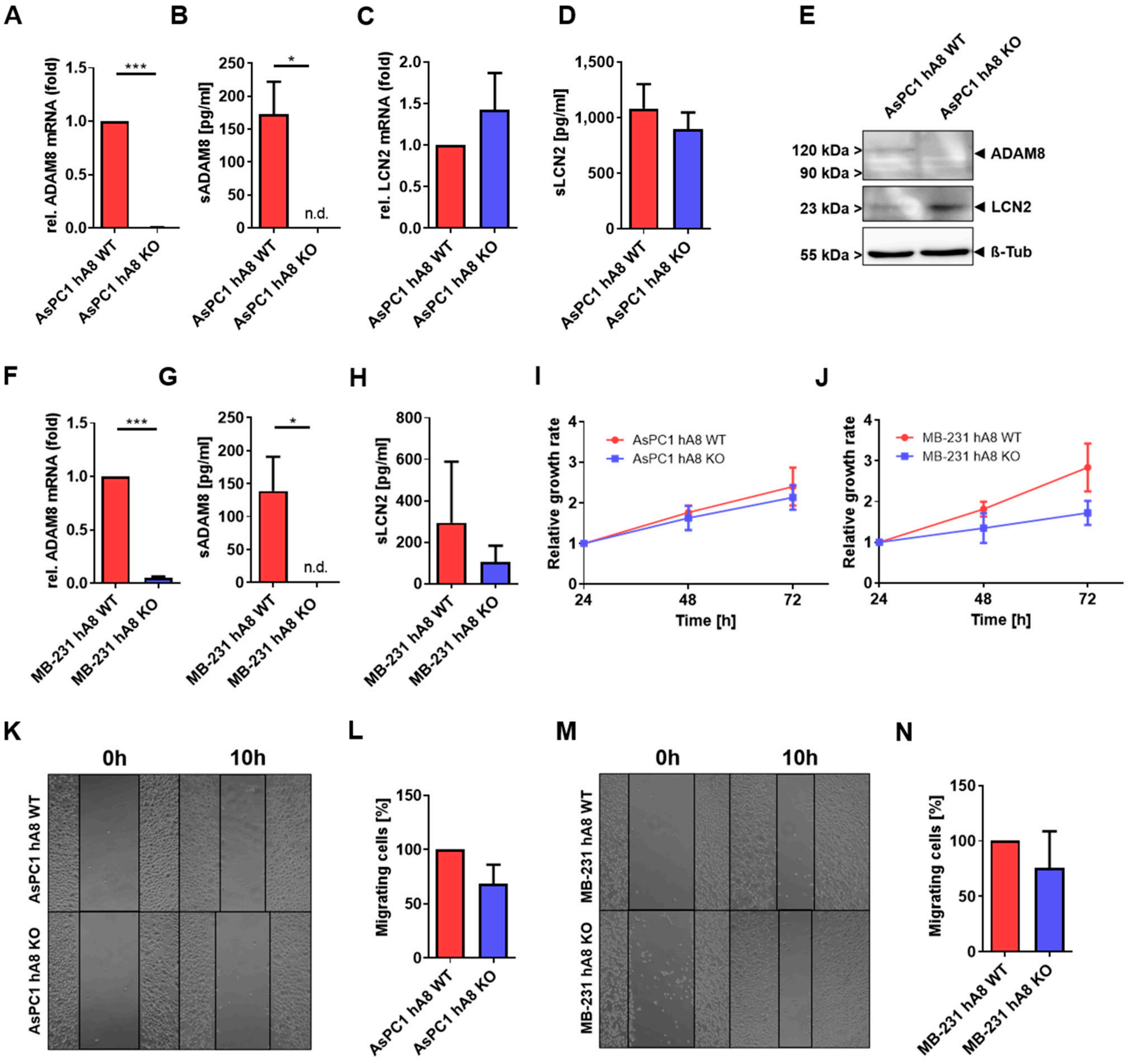

Figure S2. Effect of ADAM8 deficiency on AsPC1 and MB-231 cells. (**A**) Relative mRNA expression and (**B**) soluble protein level of ADAM8 are downregulated in AsPC1 hA8 KO cells. (**C**) Relative mRNA expression and (**D**) soluble protein level of LCN2 show no significant differences in AsPC1 hA8 WT and KO cells. (**E**) Representative immunoblot detecting ADAM8 and LCN2 in AsPC1 hA8 WT and KO cells. No ADAM8 signal was seen in KO cells, whereas the western blot of LCN2 shows a slight but not. significant upregulation of LCN2 in hA8 KO on a protein level (Quantification not shown). (**F**) Relative mRNA expression and (**G**) ELISA results show a significant decrease of ADAM8 secretion in MB-231 hA8 KO cells. (**H**) ELISA of soluble LCN2 in MB-231 cells demonstrates no significant differences in LCN2 secretion. Relative growth rates of AsPC1 hA8 WT and KO are illustrated in (**I**) and MB-231 hA8 WT and KO in (**J**). The results were not significant. Wound healing assays of AsPC1 cells (**K**) and (**L**) and of MB-231 in (**M**) and (**N**) show no significant differences between hA8 WT and KO. Data were given as mean values ± S.D. * $p < 0.05$, *** $p < 0.001$. ($n = 2$).

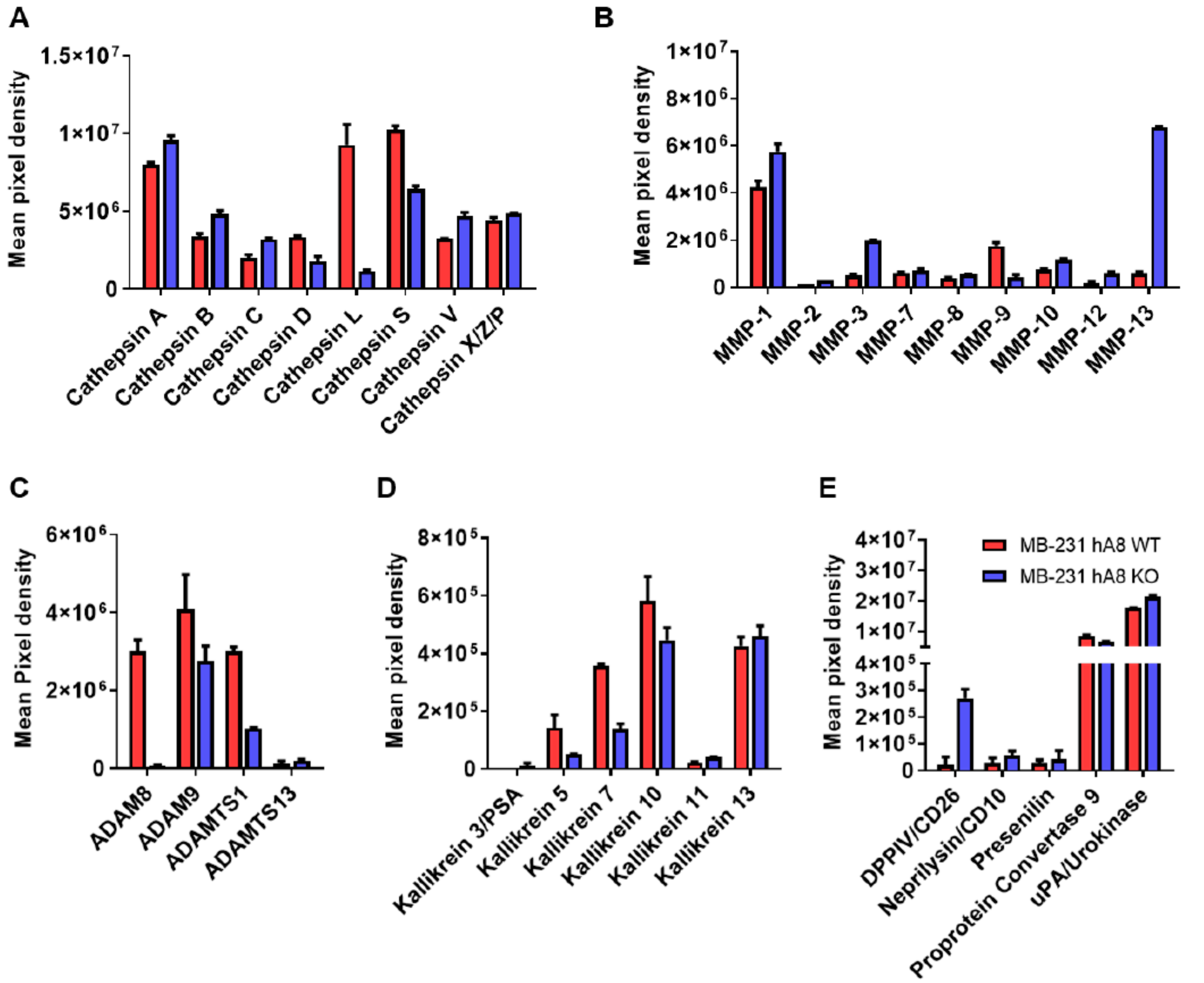

Figure S3. Human Protease Array of MB-231 hA8 WT and KO cells. The array was carried out according to the manufacturer's protocol. Mean pixel density of Cathepsin A, B, C, D, L, S, V, X/Z/P is presented in (**A**), of MMP-1, -2, -3, -7, -8, -9, -10, -12, -13 in (**B**), of ADAM8, 9, TS1, TS13 in (**C**), of Kallikrein 3/PSA, 5, 7, 10, 11, 13 in (**D**) and of DPPIV/CD26, Neprilysin/CD10, Presenilin, Proprotein Convertase 9, uPA/Urokinase in (**E**). Cathepsin L, Cathepsin S, MMP-9, ADAM8, ADAMTS1, Kallikrein 5, and Kallikrein 7 expression are decreased in MB-231 hA8 KO cells. Cathepsin A, Cathepsin B, Cathepsin C, Cathepsin V, MMP-1, MMP-3, MMP-13 show an upregulation in MB-231 hA8 KO cells. Data were given as mean values ± SD. (n = 1).

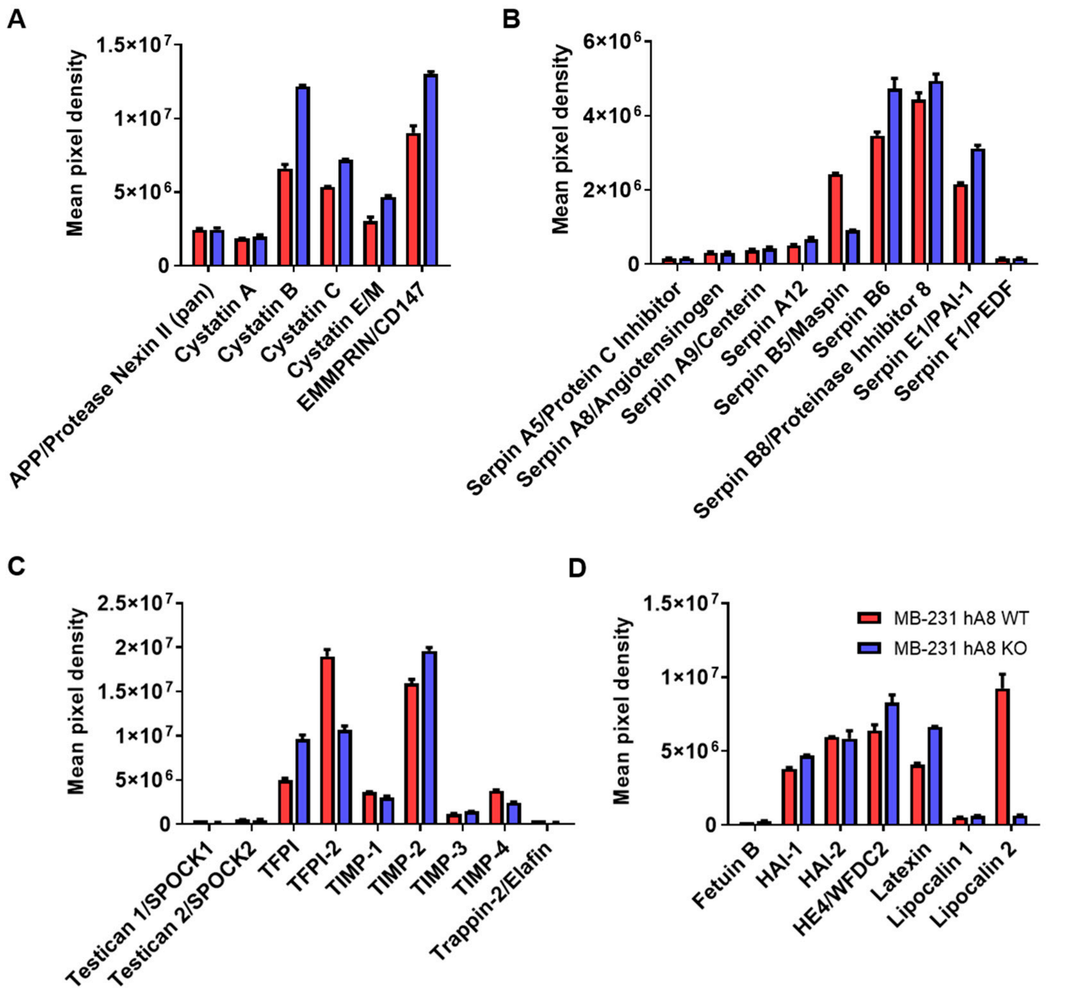

Figure S4. Human Protease Inhibitor Array of MB-231 hA8 WT and KO cells. The array was carried out according to the manufacturer's protocol. The pixel density of APP/Protease Nexin II (pan), Cystatin A, Cystatin B, Cystatin C, Cystatin E/M, EMMPRIN/CD147 are presented in (**A**), of Serpin A5/Protein C Inhibitor, Serpin A8/Angiotensinogen, Serpin A9/Centerin, Serpin A12, Serpin B5/Maspin, Serpin B6, Serpin B8/Proteinase Inhibitor 8, Serpin E1/PAI-1, Serpin F1/PEDF in (**B**), Testican 1/SPOCK1, Testican 2/SPOCK2, TFPI, TFPI-2, TIMP-1, TIMP-2, TIMP-3, TIMP-4, Trappin-2/Elafin in (**C**), Fetuin B, HAI-1, HAI-2, HE4/WFDC2, Latexin, Lipocalin 1, Lipocalin 2 in (**D**). Serpin B5/Maspin, TFPI-2, and Lipocalin- 2 are decreased in MB-231 hA8 KO cells. Cystatin B, Cystatin C, Cystatin E/M, EMMPRIN/CD147, Serpin B6, Serpin E1/PAI-1, TFPI, TIMP-2, and Latexin are upregulated in MB-231 hA8 KO cells. Data were given as mean values ± S.D. (n = 1).

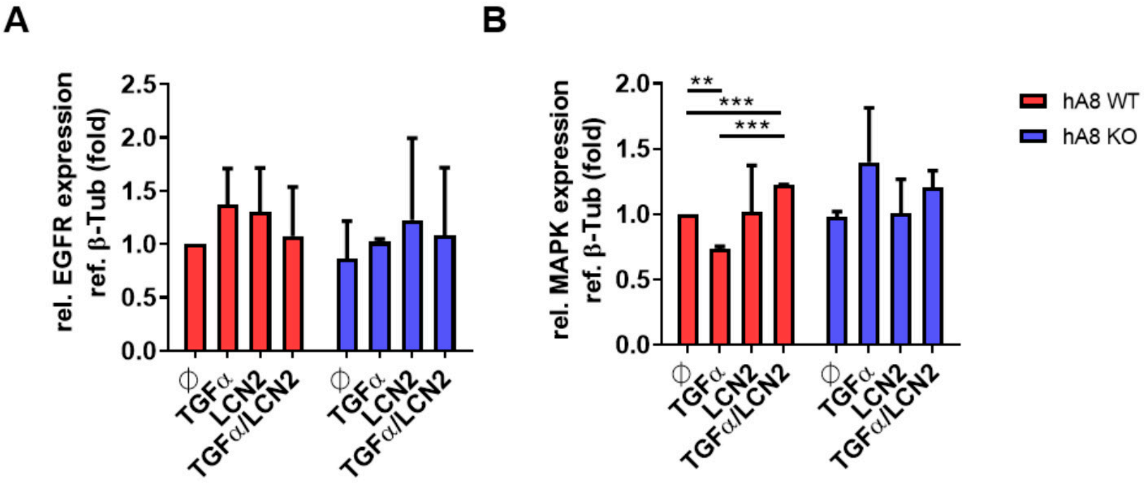

Figure S5. Relative EGFR and MAPK expression in Panc89 hA8 WT and KO cells after treatment with recombinant TGF-α (rTGF-α) and recombinant LCN2 (rLCN2). (**A**) The graph shows the quantification of EGFR expression. The presented results are not significant. (**B**) The relative MAPK expression is only significantly downregulated in Panc89 hA8 WT cells treated with 10 μM rTGF-α. The combined treatment of rTGF-α and rLCN2 increased MAPK expression in Panc89 hA8 WT cells. Data were given as mean values ± SD. ** $p < 0.01$, *** $p < 0.001$. ($n = 2$).

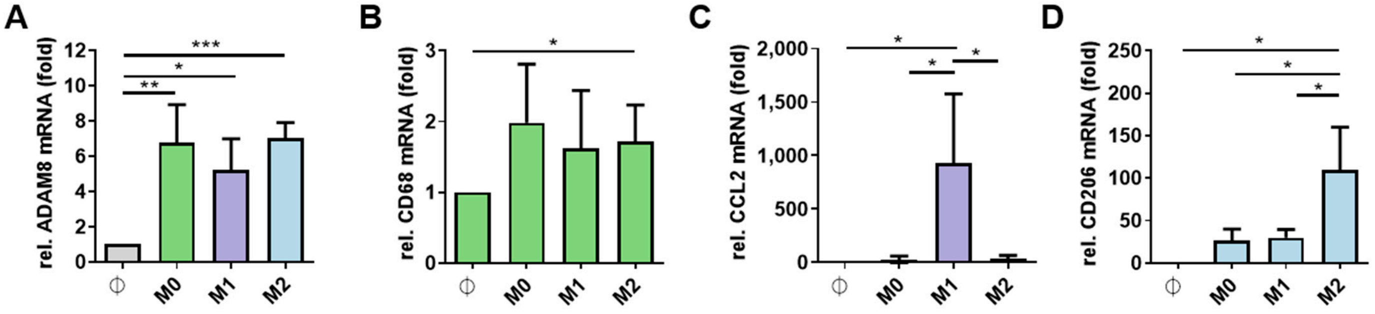

Figure S6. Differentiation and polarization of THP-1 cells. THP-1 cells were differentiated to macrophages (M0) by adding 10 ng/mL PMA to the cells. After 48 h, the medium was changed to a PMA-depleted medium, and the cells were either treated with 50 ng/mL LPS and 20 ng/mL IFNγ for M1 and 20 ng/mL IL-4 for M2 macrophages for 6 h. Isolated RNA was checked for relative ADAM8 (**A**), CD68 (**B**), CCL2 (**C**), and CD206 (**D**) mRNA expression. After differentiation and polarization, M0, M1, and M2 macrophages significantly increase relative ADAM8 mRNA expression compared to untreated THP-1 cells (O, grey). Relative CD68 mRNA expression was only considerably upregulated in M2 cells compared to O. Successful polarization of M1 and M2 was confirmed by upregulation of relative CCL2 mRNA expression in M1 macrophages and relative CD206 mRNA expression in M2 macrophages, respectively. Data were given as mean values ± S.D. * $p < 0.05$, ** $p < 0.01$, *** $p < 0.001$. ($n = 2$).

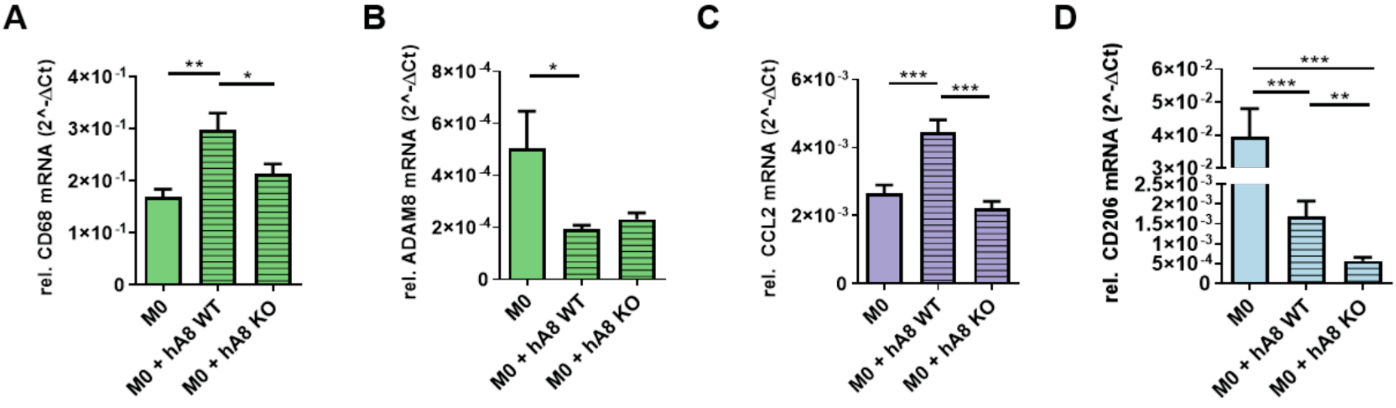

Figure S7. Gene expression changes of M0 macrophages after co-culture with Panc89 hA8 WT and KO cells. (**A**) The expression of relative ADAM8 mRNA was significantly decreased in M0 co-cultured with Panc8 hA8 WT cells, whereas relative CD68 mRNA (**B**) is increased after co-culture with Panc89 cells in an ADAM8 dependent manner. Relative CCL2 gene expression (**C**) is elevated in M0 only after coculture with Panc89 hA8 WT cells. The relative CD206 mRNA expression (**D**) is downregulated in both M0 co-cultured with Panc89 hA8 WT and KO cells. The effect is more potent after co-culture with Panc89 hA8 KO cells. Data were given as mean values ± S.D. * $p < 0.05$, ** $p < 0.01$, *** $p < 0.001$. ($n = 3$).

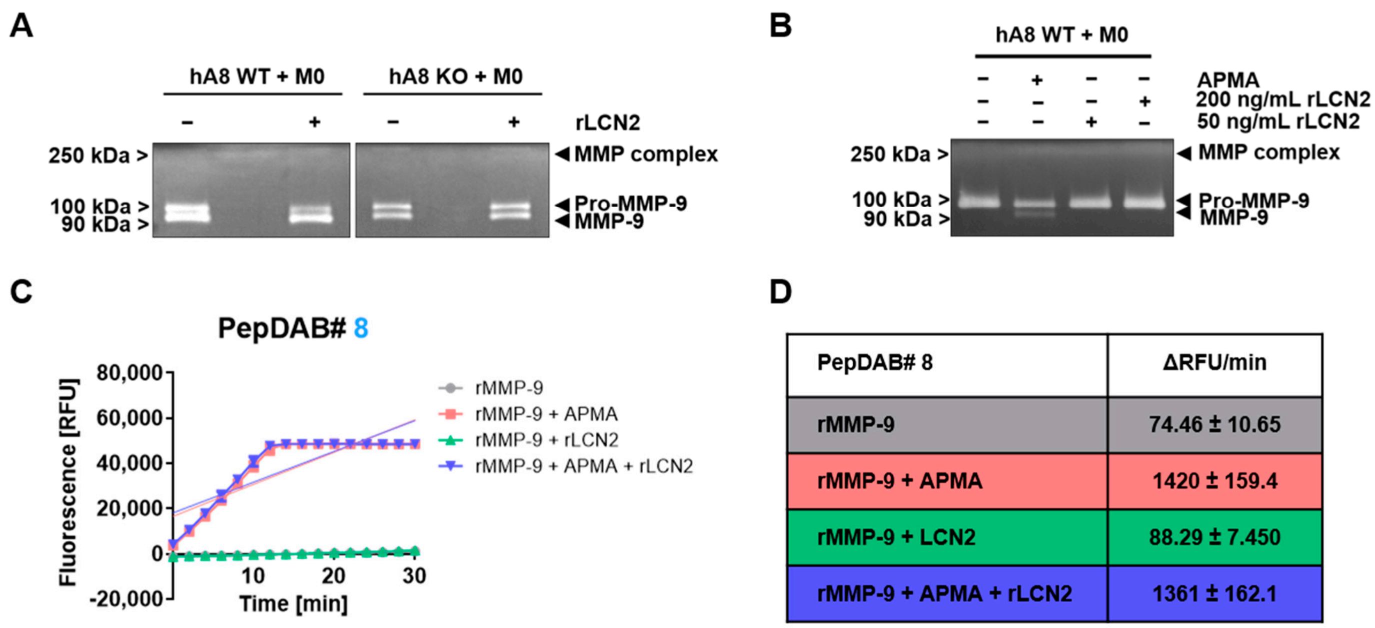

Figure S8. Impact of recombinant LCN2 on MMP-9 activity. The zymography of Panc89 hA8 WT- and KO-derived supernatants (SN) after co-culture with M0 (+ M0) is shown in (**A**). 200 ng/mL recombinant LCN2 (rLCN2) was added to the SNs just before APMA treatment to activate MMP-9. The addition of rLCN2 decreases pro-MMP-9 in SNs derived from Panc89 hA8 WT + M0, but no effects on KO + M0- derived SNs were seen. (**B**) Supernatants of Panc89 hA8 WT cells + M0 were either treated without APMA, with 0.6 mM APMA, with only 200 ng/mL rLCN2 or 50 ng/mL rLCN2. The zymography illustrates that only the addition of APMA can activate MMP-9, but not recombinant LCN2. (**C**). rMMP-9 was either untreated or treated with 0.6 mM APMA, 200 ng/mL rLCN2, or with 0.6 mM APMA. combined with 200 ng/mL rLCN2. rMMP-9 shows only activity on PepDAB# 8 after treatment with APMA (independent of rLCN2). (D) The addition of APMA to rMMP-9 increased ΔRFU/min for PepDAB# 8 from 74.46 to 1420 and, in the presence of rLCN2, from 88.29 to 1361. Data were given as mean values ± SD. (n = 1).

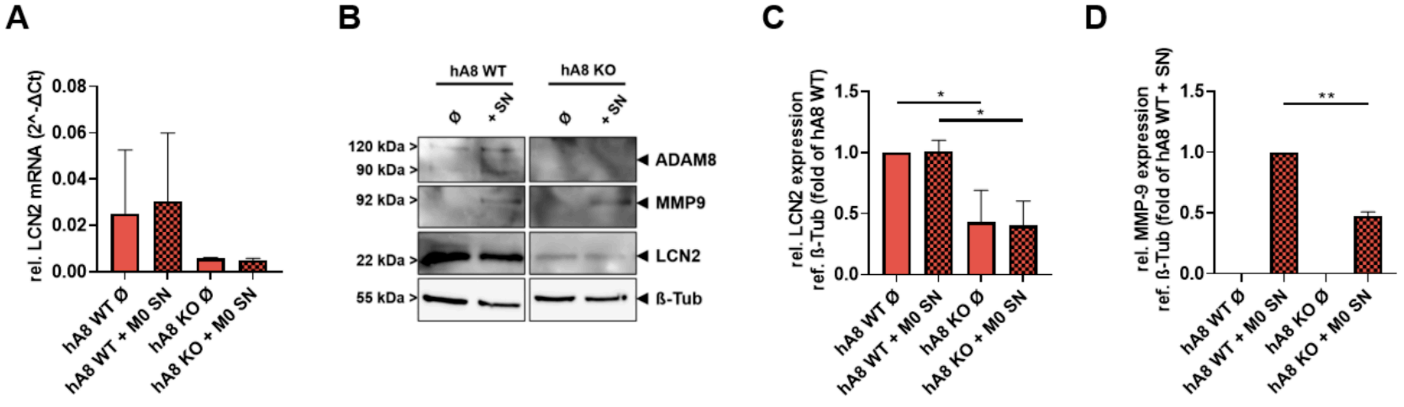

Figure S9. LCN2 and MMP-9 expression of Panc89 hA8 WT and KO cells after M0-supernatant treatment. mRNA expression of LCN2 after treatment is shown in (**A**). (**B**) displays a representative immunoblot showing the detection of ADAM8, MMP-9, and LCN2 in Panc89 hA8 WT and KO with or without treatment with M0 supernatants. Quantification of LCN2 (**C**) shows no significant difference between treated and untreated Panc89 cells, whereas the quantification of MMP-9 (**D**) shows upregulation of MMP-9 after treatment with macrophage supernatants, dependent on ADAM8 (n = 2). Data were given as mean values ± S.D. * $p < 0.05$, ** $p < 0.01$.
